# Optically detecting the edge-state of a three-dimensional topological insulator under ambient conditions by ultrafast infrared photoluminescence spectroscopy

**DOI:** 10.1038/srep16443

**Published:** 2015-11-10

**Authors:** Shun-ya Maezawa, Hiroshi Watanabe, Masahiro Takeda, Kenta Kuroda, Takashi Someya, Iwao Matsuda, Tohru Suemoto

**Affiliations:** 1 Institute for Solid State Physics, The University of Tokyo, 5-1-5 Kashiwanoha, Kashiwa, Chiba 277-8581, Japan

## Abstract

Ultrafast infrared photoluminescence spectroscopy was applied to a three-dimensional topological insulator TlBiSe_2_ under ambient conditions. The dynamics of the luminescence exhibited bulk-insulating and gapless characteristics bounded by the bulk band gap energy. The existence of the topologically protected surface state and the picosecond-order relaxation time of the surface carriers, which was distinguishable from the bulk response, were observed. Our results provide a practical method applicable to topological insulators under ambient conditions for device applications.

Three-dimensional topological insulators (TIs) are materials characterized by insulating bulk bands and a metallic surface state that results from a nontrivial topology of the bulk wave functions based on the bulk-edge correspondence^1^,^2^,^3^. The surface state has a Dirac-cone-like energy dispersion with a helical spin texture owing to strong spin-orbit interactions^1^,^2^,^3^. The helical spin structure, which suppresses electron backscattering induced by nonmagnetic impurities, provides the potential for electronic and spintronic device applications of TIs^4^,^5^. The robustness of the topologically protected surface state under exposure to air^6^,^7^,^8^ provides additional characteristics for the realization of versatile TI devices operating under ambient conditions; that is, in atmospheric air with some adsorbants on the surface. It is therefore crucial for the device applications to investigate the dynamic properties of surface carriers on TIs, particularly under ambient conditions.

Recently, the surface carrier dynamics have been explored in prototypical TIs, such as Bi_2_Se_3_ and Bi_2_Te_3_, using ultrafast time-resolved techniques to allow for distinguishing between the bulk and surface response, involving time- and angle-resolved photoemission spectroscopy (TrARPES)^9^,^10^,^11^,^12^,^13^,^14^,^15^,^16^, optical pump mid-infrared probe spectroscopy (OPMP)^17^, and optical pump THz probe spectroscopy (OPTP)^18^,^19^. TrARPES has been used to directly observe the transient electron population with high momentum and energy resolutions, which has provided knowledge about the electron-phonon scattering and bulk-surface interband transition in TIs^9^,^10^,^11^,^12^,^13^,^14^,^15^,^16^. TrARPES, however, requires ultra-high vacuum conditions to prevent surface contamination of samples, because this method has an extremely high sensitivity to the surface. In contrast, pure optical methods (OPMP and OPTP) have provided insights into the low-energy electronic transition in the surface Dirac cone even under the atmospheric air condition with a larger penetration depth than that achieved in TrARPES experiments^17^,^19^, while direct access to excited states over a wide energy range from the surface state to the bulk bands has not been realized.

Time-resolved photoluminescence spectroscopy (TrPLS) overcomes these drawbacks, owing to its lower sensitivity to the surface condition than that of TrARPES measurement and ability of wide-energy-range detection in the visible to infrared regions. Therefore, TrPLS is expected to be a practical approach to investigating the surface carrier dynamics in TIs^20^ under the ambient condition; however, no TI research with TrPLS has been reported, to the best of our knowledge.

We report the application of the infrared TrPLS technique to a TI, TlBiSe_2_, under ambient conditions (Fig. 1a). TlBiSe_2_ is known to feature the Dirac point located near the middle point of the bulk band gap energy of 0.35 eV at the 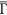 point, which is the largest value among TIs^21^,^22^,^23^. Recently, it has been demonstrated that the Fermi level of the TIBiSe_2_ crystal can be controlled by tuning its chemical composition^24^. Here, Tl_1−*x*_ Bi_1+*x*_ Se_2_ (*x* = 0.025) was studied, where the Fermi level is located near the Dirac point^24^,^25^, as shown in Fig. 1b, resulting in a low intrinsic carrier concentration in the bulk conduction and valence bands. The infrared time-resolved luminescence on the order of sub- to several picoseconds from the TlBiSe_2_ crystal was observed. At photon energies below the bulk band gap energy, the luminescence showed behavior characteristic to the gapless surface state, as distinguished from the semiconductor-like behavior above the gap energy. This observation clearly indicates the existence of the topologically protected surface state under ambient conditions.

## Results

**Figure 2:**
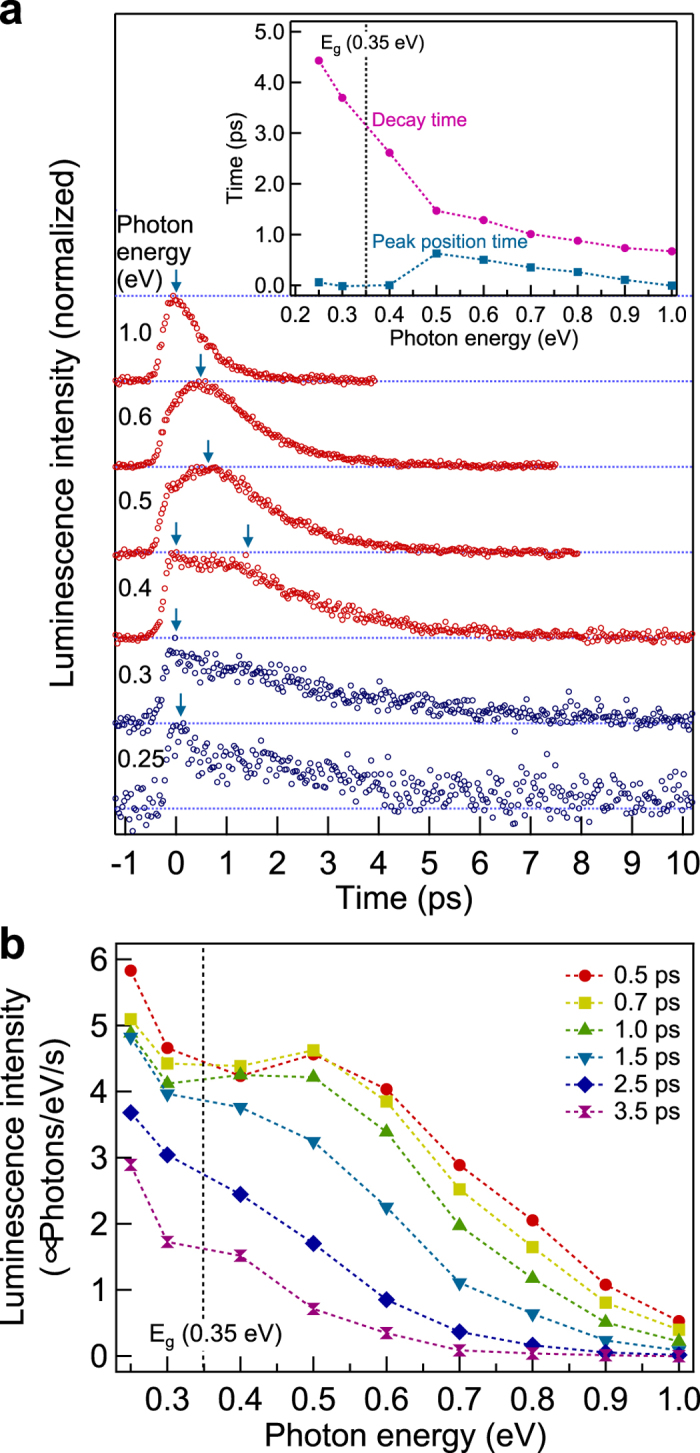
Time evolutions and spectra of the luminescence from TlBiSe_2_. (**a**) Time evolution curves of the luminescence intensities at photon energies from 0.25 eV to 1.0 eV (indicated near the left end of each curve) with 1.55 eV-photoexcitation. The curves are normalized at their own maxima and offset for clarity of display. The curves at photon energies lower than bulk band gap energy of 0.35 eV are shown in blue. The blue arrows indicate the peak position of each curve. The inset shows the peak position time and the decay time evaluated with a single exponential fitting for each decay curve. (**b**) Time-resolved luminescence spectra from 0.5 ps to 3.5 ps. Here, each spectrum is an integration within the time window of 0.2 ps that is nearly equal to the time resolution of the measurement system.

Figure 2a shows the time evolution curves of the luminescence intensities from the TlBiSe_2_ crystal at photon energies from 0.25 eV to 1.0 eV under 1.55 eV-photoexcitation. The decay time evaluated with a single exponential fitting for each curve and the peak position time, which is a measure of the rise time, are shown as functions of photon energy in the inset of Fig. 2a. The temporal profiles exhibit specifically different shapes with the photon energy. As the photon energy decreases from 1.0 eV to 0.5 eV, the peak position time becomes longer. This behavior can be explained in terms of the bulk-insulating property: The carriers are accumulated at the bottom of the bulk conduction band (or the top of the bulk valence band) owing to the large energy band gap, which obstructs phonon-mediated recombination between electrons and holes. A longer decay time at lower photon energy is attributed to a slower relaxation of the carrier population at the conduction band minimum (or the valence band maximum), reflecting the cooling dynamics of the carriers via phonon emission^26^. Conversely, when the photon energy is lower than the bulk band gap energy of 0.35 eV, distinctive temporal profiles with a fast rise time and much longer decay time (approximately 4 ps) are found at 0.25 eV and 0.3 eV. The temporal profile at 0.4 eV, which corresponds to the turning point of the temporal profiles, seems to consist of two components similar to each temporal profile at 0.3 eV and 0.5 eV.

The time-resolved luminescence spectra are derived from the data in Fig. 2a and shown in Fig. 2b. Here each spectrum is an integration within the time window of 0.2 ps that is nearly equal to the time resolution of the measurement system. The spectra at 0.5 ps and 0.7 ps exhibit a dip at the photon energy of 0.4 eV that matches the bulk band gap energy of 0.35 eV. The spectral weight moves toward lower photon energy as time elapses, reflecting the relaxation of the carrier distribution by cooling processes.

## Discussion

The distinctive change of the luminescence temporal profiles, shown in Fig. 2a, and the bimodality of the time-resolved luminescence spectra, shown in Fig. 2b, suggest that the luminescence from two different energy bands is involved. According to the band calculations^27^,^28^,^29^, no bulk state exists in the energy band gap region less than 0.6 eV, except those around the 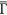 point and 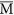 point, where each band gap energy is 0.35 eV and 0.3 eV^21^. The bulk bands around the 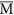 point might contribute to the luminescence at a photon energy below 0.35 eV; however, the temporal profile at the photon energy of 0.3 eV that matches the band gap energy at the 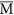 point does not exhibit a slow rise time, which is expected to arise from the bulk-insulating property. An absence of a slow rise time can be explained in terms of the gapless state that provides no barrier in phonon-mediated recombination for carriers. The temporal profiles at the photon energy below the bulk band gap energy of 0.35 eV are therefore attributed to the luminescence predominantly derived from the surface state. The decay time is consistent with a typical relaxation time of the surface state in other TIs, whose decay times are on the order of one or 10 picoseconds^9^,^10^,^11^,^12^,^13^,^14^,^15^,^16^,^17^,^18^,^19^.

The luminescence at the photon energy below the bulk band gap energy might be explained by possible trap states within the bulk band gap; however, the rise time (approximately 0.15 ps) observed at 0.3 eV is much faster than the time constant expected for the carrier trapping by defects^30^. Furthermore, the fast rising component is observed at 0.4 eV that is above the bulk band gap energy, overlapping with the slow rising component attributed to the bulk response. These facts support the interpretation that the fast rising luminescence below the bulk band gap energy originates from the surface state rather than the in-gap trap states.

**Figure 3:**
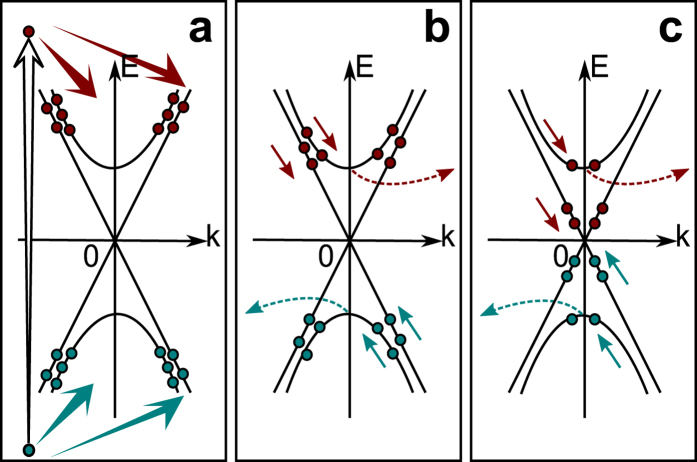
Illustration of the dynamic processes of the carriers in the rate-equation model. The bulk bands and surface state are shown by parabolas and inclined lines, respectively. Three stages of the carrier dynamics are shown in time sequence: (**a**) carrier distribution to the bulk bands and surface state (oblique arrows) just after photoexcitation (vertical arrow), (**b**) carrier cooling via phonon emission (oblique arrows) and carrier escape from the bulk bands (dashed arrows), (**c**) the subsequent carrier relaxation near the bulk band minimum or maximum and the Dirac point.

The temporal profiles are expected to be reproduced by considering the bulk and surface contributions in terms of the rate-equation model, treating each electron system separately. The rate-equation model is constructed within the photoexcitation volume composed of the surface layer and the bulk, approximated as a slab, whose thickness is assumed to be on the order of 10 nanometers referring to the optical penetration depth in a TI, Bi_2_Se_3_^10^,^31^. The bulk thickness *d* is used for the conversion of the excitation density per unit area to per unit volume in the rate equations. The energy bands relevant to the rate-equation model are illustrated in Fig. 3. The bulk bands with the bulk band gap energy *E*_*g*_ of 0.35 eV have a parabolic dispersion with an effective mass m^*^ of 0.16*m*_0_ that is a harmonic mean value of the electron effective mass 0.1*m*_0_ and hole effective mass 0.4*m*_0_ used for the theoretical calculation in ref. 32, where *m*_0_ is an electron mass. The surface state has a linear dispersion^21^ with the Fermi velocity *v*_*F*_ of 3.9 × 10^5^ m/s. The Dirac point of the surface state is set as the energy origin and the energy bands are symmetric with respect to the zero line of the energy.

Several assumptions for the carrier dynamics in the rate-equation model are used for simplicity. (i) The electrons and holes exhibit symmetric behavior and have the same temperature, which leads to the same magnitude of chemical potentials with opposite signs for the bulk electrons and holes. (ii) The chemical potential of the surface electrons is fixed to the energy origin at the Dirac point. (iii) The thermalization process before establishment of the Fermi-Dirac distribution is assumed to be very fast and is not explicitly considered in the present model^12^.

The excitation and relaxation processes of the carriers are shown in Fig. 3. A Gaussian-shaped pump pulse *G*(*t*) = *G*_0_ exp(−(*t* − *t*_0_)^2^/*τ*^2^) excites the carriers. The carriers, which instantaneously relax to the energy level *E*_*i*_ losing a part of the energy gained from the excitation, are distributed to the bulk bands and surface state in a ratio of *a* to 1 − *a* (Fig. 3a). The carriers in the bulk bands and surface state are separately relaxed via phonon emission with the coupling constants *k*_*b*_ and *k*_*s*_ (Fig. 3b), and gradually accumulate near the bottom or top of each band (Fig. 3c). Some of the carriers escape from the bulk bands with the time constant *τ*_*b*_. The time derivatives of the total energy per unit volume of the bulk electron system (*U*_*b*_), the total energy per unit area of the surface electron system (*U*_*s*_) and the bulk electron density per unit volume (*N*_*b*_) are given by the following rate equations:


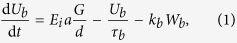



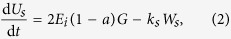



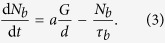


Here, *W*_*b*,*s*_ denote the phonon interaction with the bulk and surface electrons:


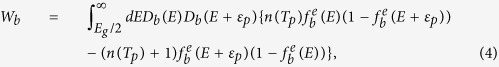



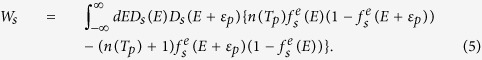


*D*_*b*,*s*_ denote the density of states for the bulk and surface electrons, respectively. 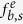 represent the Fermi-Dirac distribution for the bulk and surface electrons. Einstein’s model is used to describe the phonon distribution; *n*(*T*_*p*_) denotes the Bose distribution for the phonon with the phonon energy *ε*_*p*_ fixed to 23 meV, referring to the highest value of the phonon energy in Bi_2_Se_3_^10^,^33^. The phonon temperature *T*_*p*_ is fixed to room temperature of 300 K, assuming the phonon system does not obtain energy from the electron systems.

The luminescence intensity *I*(*E*_*l*_) at photon energy *E*_*l*_, treating the radiative recombination in the bulk bands and surface state separately, is given by:


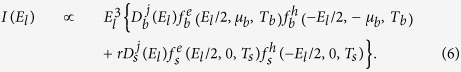


Here, *r* is a weighting factor. 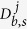 are the joint density of states for the bulk bands and surface state, respectively. 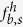 represent the Fermi-Dirac distribution for the bulk and surface holes. The chemical potential of the bulk electrons (*μ*_*b*_) and the bulk electron temperature (*T*_*b*_) are derived from the bulk electron density and the total energy of the bulk electron system. The surface electron temperature (*T*_*s*_) is also derived from the total energy of the surface electron system. The detailed expressions for the rate equations can be found in Supplementary Information.

**Figure 4:**
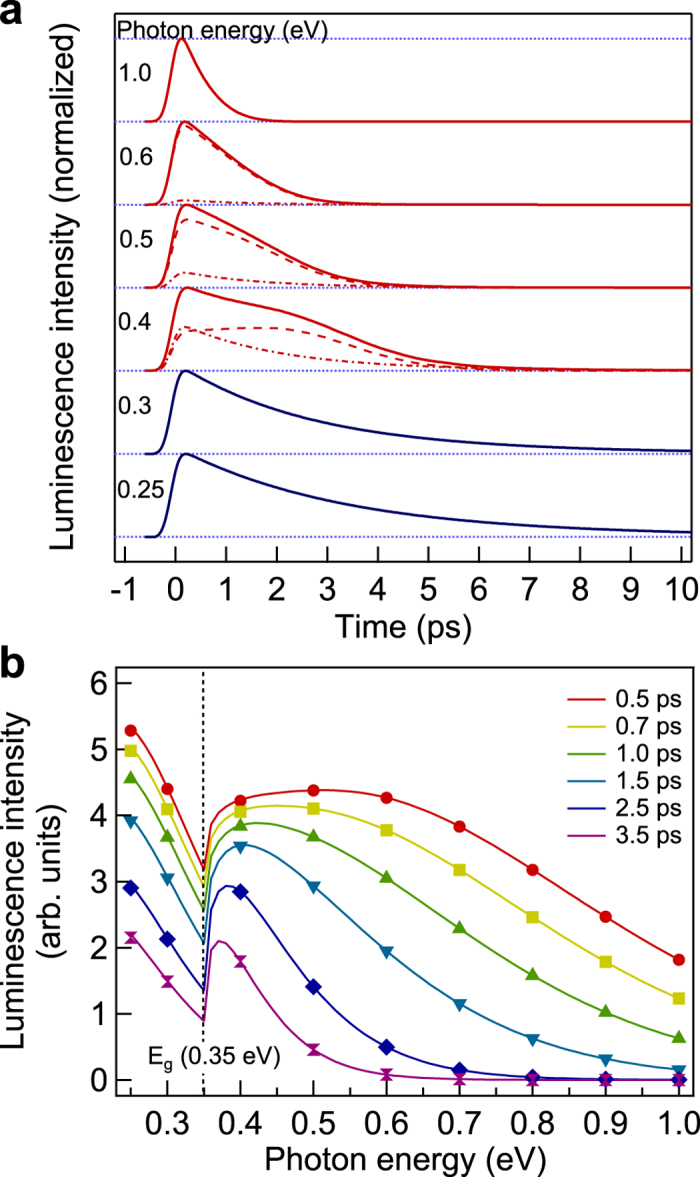
Calculated time evolutions and spectra of the luminescence. (**a**) Calculated time evolution curves of the luminescence intensities at photon energies from 0.25 eV to 1.0 eV. Dashed and chain curves are shown for the luminescence from the bulk band and surface state, respectively, at 0.4 eV, 0.5 eV and 0.6 eV. The curves are normalized at the maxima of the summation of the two contributions and offset for clarity of display. The curves at photon energies lower than the bulk band gap energy of 0.35 eV are shown in blue. (**b**) Calculated time-resolved luminescence spectra from 0.5 ps to 3.5 ps (solid curves with symbols).

Figure 4a,b show the calculation results of the time evolution curves of the luminescence intensities and the time-resolved luminescence spectra, for the following parameters: *G*_0_ = 1.3 × 10^25^ cm^−2^ s^−1^, *τ* = 60 fs, *E*_*i*_ = 0.45 eV, *a* = 10/11, *k*_*b*_ = 6.7 × 10^−10^ eV^2^cm^3^ s^−1^, *k*_*s*_ = 1.9 × 10^−3^ eV^2^cm^2^ s^−1^, *τ*_*b*_ = 1.8 ps and *r* = 4.8. The central time of the Gaussian-shaped pump pulse *t*_0_ is set to −0.1 ps to fit the peak positions on the time axis, and the calculated temporal profiles are convoluted with a Gaussian function with FWHM of 0.3 ps as the instrumental function. The model calculation reproduces the tendency that the temporal profile exhibits a longer peak position time at lower photon energy (Fig. 4a). The bulk and surface contributions are separately shown with the dashed and chain curves for 0.4 eV to 0.6 eV. At 0.4 eV, the surface contribution is comparable to that of the bulk. The surface contribution is dominant around the time origin owing to the short rise time, while the bulk contribution becomes dominant around 2–3 ps owing to the long rise time reflecting the bulk-insulating property. Above 0.5 eV, the surface contribution is negligibly small, because the surface electron temperature is much lower than that of the bulk electron (see Supplementary Fig. S1a). The model calculation also reproduces the spectral dip at an early time and the spectral weight shift toward the lower photon (Fig. 4b). The spectral dip results from the summation of the bulk and surface contributions (see Supplementary Fig. S2).

The agreement of the model calculations with the experimental results provides a plausible interpretation for the luminescence from a TI TlBiSe_2_ in terms of the bulk and surface contributions. Previous researches on TIs have proposed that the bulk conduction band acts as a charge reservoir for the surface state, which induces slow rise^15^ and long-lived relaxation^10^ of the surface population through transfer of the electrons. However, the luminescence from the surface state in TI TlBiSe_2_ exhibits a much faster rise time than that of the bulk band near the band minimum. In other words, the bulk-surface coupling effect is not noticeable in our experiment, if at all. The slow decay time of the luminescence is attributed to the relaxation of the surface electron temperature in our model calculations rather than the delayed transfer of the electrons from the bulk band (see Supplementary Fig. S1a). The insignificance of the bulk-surface coupling effect could possibly be explained by the difference between the energy dispersion of the surface state under the present ambient condition and the ultra-high vacuum condition in photoemission spectroscopy.

In conclusion, the infrared TrPLS technique was applied to the investigation of the surface state in a TI, TlBiSe_2_, under ambient conditions. The time-resolved luminescence was observed for the first time in TIs in the near- and mid-infrared regions that is wide enough to observe the comprehensive behavior of the carrier relaxation both in bulk and surface states. A distinctive shape change of the temporal profiles with the photon energy bordering on the bulk band gap energy was successfully interpreted as the alternation in the luminescence origin between the bulk-insulating states and the gapless state. This observation clearly showed the existence of the topologically protected surface state even under ambient conditions and revealed the picosecond-order relaxation time of the surface carriers, which is distinguishable from the bulk response with the photon energy. Our results present the ability of the infrared TrPLS technique as a novel approach to the dynamic properties of the surface carriers in TIs toward their application in electronic and spintronic devices operating in an ambient environment.

## Methods

Ultrafast infrared photoluminescence spectroscopy was performed with the up-conversion technique^34^,^35^,^36^,^37^ in air at room temperature. The TlBiSe_2_ sample was excited using 70 fs pulses at a wavelength of 800 nm (1.55 eV) from a Ti:sapphire regenerative amplifier operating at a repetition rate of 200 kHz. The spot size on the sample was approximately 300 *μ*m in diameter and the fluence was estimated to be 0.34 mJcm^−2^. The visible light was generated by sum-frequency mixing of the infrared luminescence light and the gating pulses at 1.55 eV in an optical nonlinear crystal, LiIO_3_, and detected by a photomultiplier tube coupled with a double grating monochromator. The time resolution estimated by autocorrelation of the pump pulse reflected by the sample and the gating pulse was 170 fs. The energy resolution was approximately 70 meV. The up-conversion measurement system had a high sensitivity for luminescence photons between 0.23 eV and 1.3 eV^35^,^37^. The spectral sensitivity of the system was calibrated using sum-frequency signals between light from a tungsten lamp with a sapphire window and the gating pulses at 1.55 eV.

## Additional Information

**How to cite this article**: Maezawa, S. *et al.* Optically detecting the edge-state of a three-dimensional topological insulator under ambient conditions by ultrafast infrared photoluminescence spectroscopy. *Sci. Rep.* **5**, 16443; doi: 10.1038/srep16443 (2015).

## Supplementary Material

Supplementary Information

## Figures and Tables

**Figure 1:**
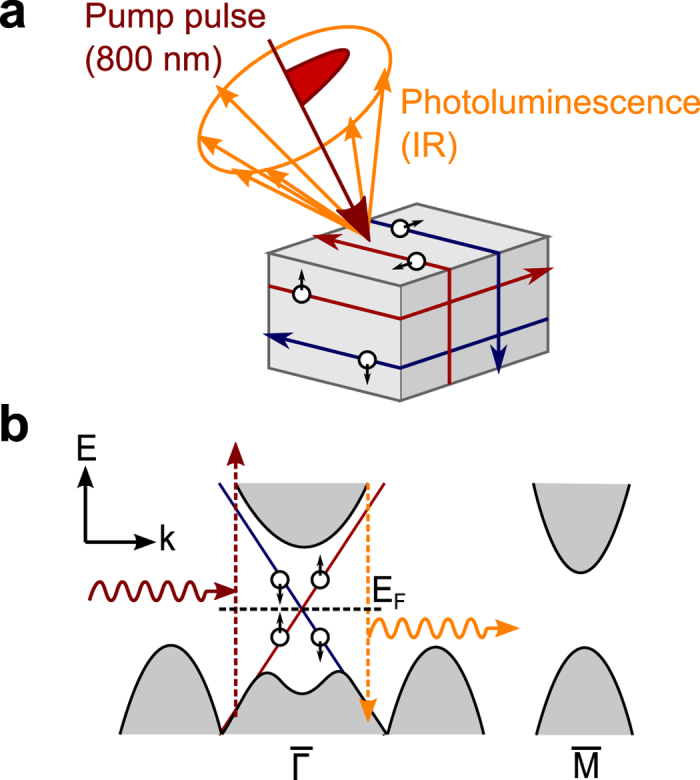
Concept of the experiment. (**a**) Schematic of the infrared photoluminescence measurement at a pulse excitation of 1.55 eV (800 nm). The momentum-spin locked currents along the surfaces are shown by red and blue arrows. (**b**) Schematic band structures of the bulk bands and surface state near 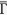 and 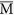 points for TlBiSe_2_, with optical pumping (red arrow) and infrared luminescence (orange arrow).
