# Supplementary material for: Optically detecting the edge-state of a three-dimensional topological insulator under ambient conditions by ultrafast infrared photoluminescence spectroscopy
Source: Sci Rep. 2015 Nov 10;5:16443. doi: 10.1038/srep16443 (PMC4639728; doi:10.1038/srep16443)
Supplement: Supplementary Information [file srep16443-s1.pdf]

# Supplementary Information for "Optically detecting the edge-state of a three-dimensional topological insulator under ambient conditions by ultrafast infrared photoluminescence spectroscopy"

Shun-ya Maezawa*, Hiroshi Watanabe, Masahiro Takeda, Kenta Kuroda, Takashi Someya, Iwao Matsuda and Tohru Suemoto

*Institute for Solid State Physics, The University of Tokyo, 5-1-5 Kashiwanoha, Kashiwa, Chiba 277-8581, Japan*

## 1. Expressions for the rate equations

In the rate-equation model, as shown in Fig. 3, the bulk bands with the bulk band gap energy $E_g$ and the surface state are assumed to have a parabolic dispersion with the effective mass $m^*$ and a linear dispersion with the Fermi velocity $v_F$. For simplicity, the Dirac point of the surface state is set as the energy origin and the energy bands are assumed to be symmetric with respect to the zero line of the energy, which enables the electrons and holes to exhibit symmetric behavior.

The density of states for the bulk electrons ($D_b$) and surface electrons ($D_s$) are given by:

$$D_b(E) = \frac{1}{2\pi}\left(\frac{2m^*}{\hbar^2}\right)^{\frac{3}{2}}\left(E-\frac{E_g}{2}\right)^{\frac{1}{2}}, \tag{1}$$

$$D_s(E) = \frac{1}{2\pi(\hbar v_F)^2}|E|. \tag{2}$$

The relationships between the bulk electron density per unit volume ($N_b$), the total energy per unit volume of the bulk electrons ($U_b$), the chemical potential of the bulk electrons ($\mu_b$) and the bulk electron temperature ($T_b$) are given by:

$$N_b = \int_{E_g/2}^{\infty} dE D_b(E) f_b^e(E,\mu_b,T_b), \tag{3}$$

$$U_b = \int_{E_g/2}^{\infty} dE D_b(E) f_b^e(E,\mu_b,T_b)E, \tag{4}$$

where $f_b^e$ denotes the Fermi-Dirac distribution for the bulk electrons. The relation between the total energy per unit area of the surface electrons ($U_s$) and the surface electron temperature ($T_s$), derived under the condition that the chemical potential of the surface electrons is fixed to zero, is given as follows in a similar way to graphene[S1]:

$$U_s = \frac{3\zeta(3)}{2\pi(\hbar v_F)^2}(k_B T_s)^3, \tag{5}$$

*E-mail: maezawa@issp.u-tokyo.ac.jp

where $\zeta$ is the Riemann zeta function and $k_B$ is the Boltzmann constant.

The joint density of states for the bulk electrons ($D_b^j$) and surface electrons ($D_s^j$) are given by:

$$D_b^j(E_l) = \frac{1}{2\pi}\left(\frac{2m^*}{\hbar^2}\right)^{\frac{3}{2}}\left(\frac{E_l}{2}-\frac{E_g}{2}\right)^{\frac{1}{2}}, \quad (6)$$

$$D_s^j(E_l) = \frac{1}{2\pi(\hbar v_F)^2}\frac{E_l}{2}, \quad (7)$$

where $E_l$ denotes the photon energy of the luminescence.

# 2. Calculation results

Figure S1a shows the calculated time evolutions of the bulk electron temperature (solid curve) and surface electron temperature (dashed curve). Each temperature immediately reaches the maximum value around the time origin. In this calculation, the maximum value of the bulk electron temperature is much higher than that of the surface electron temperature. This result can be explained in terms of the different nature of the band structures: The bulk conduction and valence bands are separated by the band gap, while the surface state has a gapless structure. The bulk electron population is formed in the bulk conduction band with high-energy electrons excited by the pump pulse $G$. In this case, the maximum value of the bulk electron temperature is mainly determined by the value of $E_i$, and it does not depend on the excitation density, because the deposited energy is proportional to the created electron number. Here $E_i$ is the energy of the electrons initially distributed in the bulk conduction band. In contrast, in the gapless system, the energy deposited by the incident photon is immediately shared by many electrons produced near the Fermi energy to establish the Fermi-Dirac distribution appropriate for the temperature determined by the deposited energy. The maximum value of the surface electron temperature is then a monotonically increasing function of the excitation density and it does not reflect the excitation photon energy directly. Therefore, the initial electron temperature in the surface state can be very low under a moderate excitation density. After reaching the maximum value, each temperature decreases toward room temperature owing to the interaction with the phonon. Figure S1b shows the calculated time evolution curve of the bulk electron density per unit volume.

Figure S2 shows the calculated time-resolved luminescence spectra from 0.5 ps to 3.5 ps. The bulk and surface contributions are separately shown with the dashed and chain curves, respectively. In the time-resolved luminescence spectra at 0.5 ps and 0.7 ps, the intensities at 0.4 eV are slightly lower than those at 0.3 eV and 0.5 eV, reproducing the experimental results (Fig. 2b). As time elapses, the whole spectral weight moves toward lower photon energy, dominated by the spectral weight shift of the bulk contribution.

# References

S1. Sun, D. *et al.* Hot carrier cooling by acoustic phonons in epitaxial graphene by ultrafast pump-probe spectroscopy. *Phys. Status Solidi C* **8**, 1194–1197 (2011).

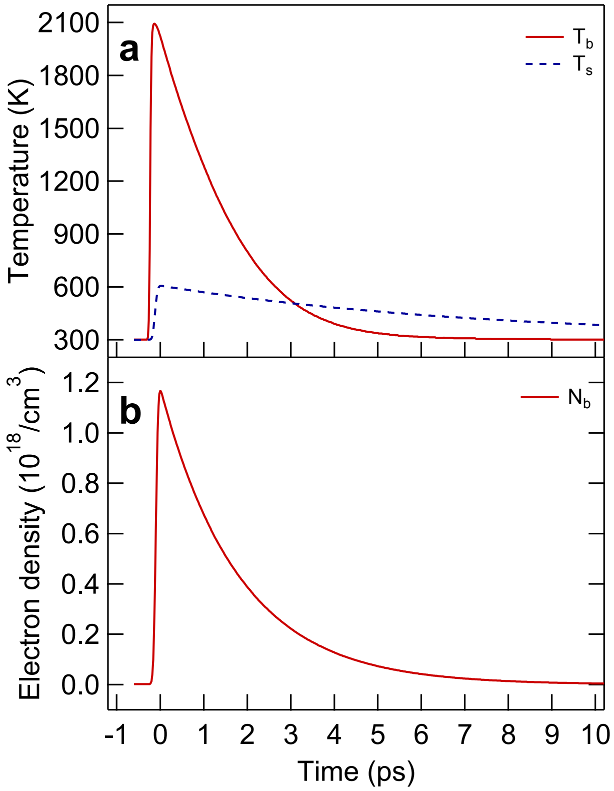


**Figure S1.** (a) Calculated time evolutions of the bulk electron temperature (solid curve) and surface electron temperature (dashed curve). (b) Calculated time evolutions of the bulk electron density per unit volume.

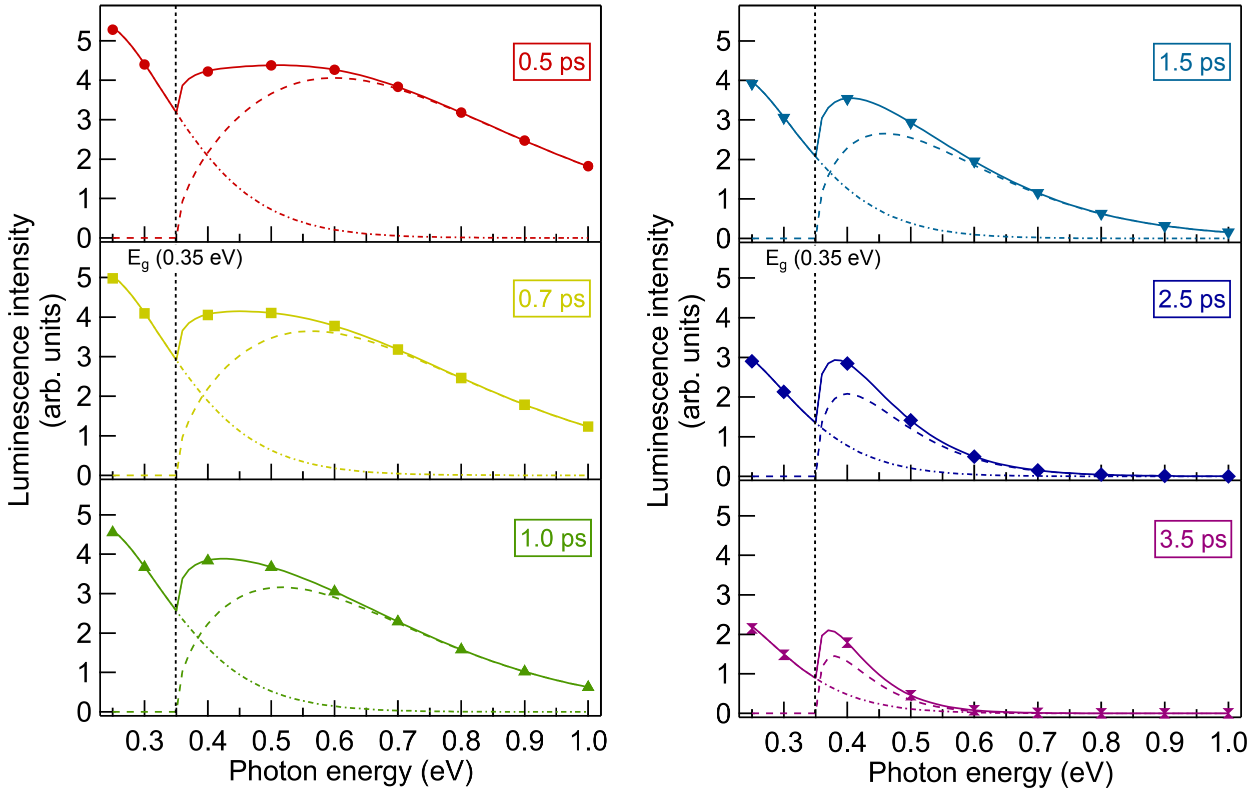


**Figure S2.** Calculated time-resolved luminescence spectra from 0.5 ps to 3.5 ps. Dashed and chain curves correspond to the luminescence from the bulk band and surface state, respectively. Solid curves with symbols correspond to the summation of the two contributions.
